# Feed Restriction Improves Lipid Metabolism by Changing the Structure of the Cecal Microbial Community and Enhances the Meat Quality and Flavor of Bearded Chickens

**DOI:** 10.3390/ani12080970

**Published:** 2022-04-08

**Authors:** Jinling Ye, Shouqun Jiang, Zhonggang Cheng, Fayuan Ding, Qiuli Fan, Xiajing Lin, Yibing Wang, Zhongyong Gou

**Affiliations:** 1 Institute of Animal Science, Guangdong Academy of Agricultural Sciences, Guangzhou 510640, China; yejinling@gdaas.cn (J.Y.); chengzhonggang@yeah.net (Z.C.); dingfayuan@163.com (F.D.); fanqiuli_829@163.com (Q.F.); linxiajing728@sohu.com (X.L.); wangyibing77@163.com (Y.W.); yozhgo917@163.com (Z.G.); 2 State Key Laboratory of Livestock and Poultry Breeding, Guangzhou 510640, China; 3 Key Laboratory of Animal Nutrition and Feed Science in South China, Ministry of Agriculture and Rural Affairs, Guangzhou 510640, China; 4 Guangdong Provincial Key Laboratory of Animal Breeding and Nutrition, Guangzhou 510640, China

**Keywords:** Bearded chickens, feed restriction, meat quality, flavor, cecal microbial community

## Abstract

**Simple Summary:**

With the development of intensive feeding, the increased cost of feeding and the excessive fat deposition affecting carcass and meat quality in full-fed broiler chickens have attracted more attention from poultry farmers and consumers. Feed restriction (FR) has been adopted to tackle these problems. The present study demonstrated that 80% feed intake showed a significant role in improving lipid metabolism and enhancing the meat quality and flavor of Bearded chickens. Additionally, this study confirms that the major mechanisms are the activation of the calproteinase system and the regulation of the structure of cecal microflora.

**Abstract:**

Excessive fat deposition in full-fed Bearded chickens does not only reduce carcass yield but also causes consumer rejection of meat. Feed restriction (FR) is an effective method to save on feed cost, reduce carcass fat deposition, and improve meat quality. A total of 560 150-d Bearded chickens were randomly divided into seven groups (each with eight replicates of ten birds) for 40 days. The control group was fed with the basal diet ad libitum (CON), and the other six groups were fed with 90% of the feed intake (90% FI), 80% FI, 70% FI, 90% metabolizable energy (90% ME), 80% ME, and 70% ME of the CON, respectively. Compared to the CON group, FR increased meat yield, but the total weight of the Bearded chickens was slighter; 80% FI and 70% ME improved the relative lipid metabolism indices of chickens, especially the levels of triglycerides and total cholesterol in the plasma and liver (*p* < 0.05), and decreased calpastatin activity in the breast muscle (*p* < 0.05). Additionally, 16S rRNA sequencing of cecal microbial community indicated that an increase in the abundance of *Hydrogenoanaerobacterium* and *Bacteroides plebeius* was observed in the 80% FI group (*p* < 0.05), and an enrichment in *Olsenella*, *Catabacter*, and *Lachnospiraceae* were observed in the 70% ME group (*p* < 0.05) compared to the CON group. Moreover, compared to the CON group, the L * value of the breast muscle significantly decreased, and a * value significantly increased in the 80% FI group (*p* < 0.05). Notably, the concentrations of threonine, lysine, aspartic acid, glutamic acid, proline, and arginine and the activity of calpain in breast muscle increased in the 80% FI group more than in the CON group (*p* < 0.05), while valine, isoleucine, leucine, phenylalanine, lysine, alanine, tyrosine and proline decreased in ME restriction groups (*p* < 0.05). Taken together, our results indicated that 80% FI could improve lipid metabolism by changing the structure of the cecal microbial community, and the meat quality and flavor of the Bearded chickens in 80% FI group was improved with a promoted meat color score, flavor substances, and the calproteinase system.

## 1. Introduction

Bearded chickens in China have the appearance characteristic of “three yellow and one beard” and a poorer growth rate and feed conversion rate than commercial birds. However, Chinese indigenous chickens are famous genetic resources in the world for their early maturity, good meat quality and strong resistance to disease. During the last few years, slow-growing broilers, represented by Huiyang Bearded chickens, have been favored by consumers. Nevertheless, the excessive fat deposition caused by feeding freely restricted the Bearded chickens from expanding to the industrial scale [1]. Fortunately, feed restriction (FR) has been shown to be an effective method to manipulate lipid metabolism with related enzymes to reduce fat deposition and improve feed efficiency [2,3,4,5]. Furthermore, reducing feed intake (FI) or limiting calories in a diet can effectively save farming costs. Early studies reported that the FR was divided into two types: quantitative (limited daily feed supply) and qualitative (finite diet nutrient dilution) restriction [5,6], and these different treatments may not achieve the same results.

Both the intensity and stage of restricted feeding affected the carcass performance and meat quality of the broilers [7,8]. A previous study demonstrated that the final body weight (FBW), carcass, and breast of the Lohman strain were higher than those of the Cobb, while the wings and brisket of the Cobb were higher than the Lohman’s after FR [9]. Most studies have focused on early restriction diets for commercial broilers (Ross 308 [3], Cobb [10,11] and Hubbard [12]) and found that early feed restriction programs were effective in reducing abdominal and carcass fat. Nevertheless, whether the carcass performance of the poultry can catch up with that of the unrestricted feeding group depends on compensatory growth [6].

Interestingly, quantitative feed restriction had significant effects on the microbiota of the ceca in broilers, except for lactic acid bacteria [13]. In addition, it has been indicated that intestinal microorganisms play an important role in regulating fat metabolism in broiler chickens [14,15]. It was found that the cecal microbiota in chickens contributed significantly to fat deposition, accounting for 21% of the variability in abdominal fat mass after adjustment for host genetic effects [16].

Therefore, to expand the high-quality breed chicken industry and provide more healthy meat, a study on FR for Bearded chickens before marketing is necessary and important. In particular, whether FR is beneficial to 150-day-old Bearded chickens or causes chronic stress requires further exploration. Our study aims to evaluate the effect of FR on the meat quality, flavor, and cecal microbial community of Bearded chickens in the fattening period so as to determine the appropriate dietary nutrition levels. We hope that this research will provide a scientific basis for chicken’s breeding and bring more economic benefits to feed companies and farmers.

## 2. Material and Methods

### 2.1. Animals and Experimental Design

The experimental protocol was approved by the Animal Care Committee of the Institute of Animal Science, Guangdong Academy of Agriculture Science, Guangzhou, P. R. China, with the approval number GAASISA-2019-020. In this work, 560 150-day-old Bearded chickens (female) with similar average initial body weights (1414.41 g) were randomly allotted seven treatments, each treatment with eight replicates and ten chickens per replicate, in a completely randomized design. The control group was fed the basal diet ad libitum (CON), and the other six groups were fed with 90% of the feed intake (90% FI), 80% FI, 70% FI, 90% metabolizable energy (90% ME), 80% ME, and 70% ME of the CON, respectively. The experiment used corn-soybean meal basal rations, and the basic dietary nutrition level refers to NY/T 3645-2020 “Nutrient Requirements for Yellow-feathered Chickens,” newly compiled by the Institute of Animal Science, Guangdong Academy of Agricultural Sciences, and scientifically formulated experimental diets. The specific experimental ration formula and nutritional levels are shown in Table 1.

Birds were weighed per replicate at the beginning (d 150) and the end of the experiment (d 190). The average daily feed intake (ADFI) was calculated using records of daily feed consumption on a pen replicate basis. The mortality of each treatment group was examined daily, and the number of dead birds was recorded and weighed to adjust the feed intake calculations. The average daily gain (ADG) and feed-to-gain ratio (FCR) were calculated from 150 to 190 d.

### 2.2. Sample Collection

On day 190, two chickens with close to average FBW were selected for each replicate and weighed immediately before slaughter. Heparinized blood samples were collected from the brachial vein; plasma samples were separated at 4 °C with 1200× *g* centrifuged for 10 min, and the plasma distribution was stored at −80 °C. Then the birds were stunned by electric shock and exsanguinated and weighed after removing feathers, feet, and beak shells to obtain the carcass weight. Then, the breast muscles, thigh muscles, and abdominal fat (including fat around the musculature and stomach) were divided and weighed to calculate the percentage of breast muscle, thigh muscle, and abdominal fat based on the carcass weight. The breast muscle and liver were rapidly harvested from the same region on the right, quickly frozen in liquid nitrogen, and stored at −80 °C for RNA extraction to determine related gene expression and biochemical analysis. The remaining breast muscle of the right side was used for testing the histological character of muscle fiber, intramuscular fat (IMF), inosine-5′-monophosphate (IMP), and free amino acid, and the left breast muscle was used to measure the relative indices of meat quality. Meanwhile, the remaining liver was used to measure fat content. The digest of the cecum was collected and frozen in liquid nitrogen and then stored at −80 °C for later bacterial DNA isolation and further analysis.

### 2.3. Meat Quality and Flavor Substance

At 45 min or 24 h after slaughter, the pH was measured by inserting three electrodes into the left breast muscle using a portable pH meter (HI 8424C, Beijing Hanna Instruments Science & Technology, Beijing, China).

The color coordinates (L *, a *, and b *) were measured using a colorimeter (CR-410, Minolta Co., Ltd., Suita, Osaka, Japan). Based on the domestic and foreign literature reports, our research group established the technical procedures for evaluating the muscle color of yellow-feathered broiler chickens. Firstly, the main instrument used for measurement was the CR410 chromaticity meter, which was corrected with a standard white tile before use. Secondly, the assessment time was between 30 and 45 min after slaughter. Thirdly, the determination site was the inner muscle of pectoralis major near the bone so as to avoid the influence of the discoloration of the pectoralis surface caused by scalding on the determination results. Fourthly, the detailed steps of the measurement were to lay the whole stripped pectoralis major muscle flat on a white enamel plate, and then take three points along the midline of the long axis of the pectoralis major muscle from thick to thin, and then measure the surface of the muscle near the bony side. Finally, the average value of the three measurements was calculated as the final result.

The muscle samples were suspended on steel wire hooks and placed in a sealed plastic bag without contact, then wiped and re-weighed 24 h later at 4 °C, following the procedures of Cui et al. [17].

The Warner–Bratzler shear force was determined with samples heated at 85 °C until the internal temperature was 75 °C and monitored with a digital thermometer using an Instron Universal Mechanical Machine (Instron Model 4411, InstronCorp., Canton, MA, USA).

The IMF content was assessed using the Soxhlet petroleum-etherextraction procedure; the IMP content was measured through high-performance liquid chromatography (Agilent 1200; Agilent Technologies, Santa Clara, CA, USA), with IMP disodium salt hydrate (Sigma-Aldrich, St. Louis, MO, USA) as the internal standard; the composition of the amino acids was determined on an automatic amino acid analyzer (L-8900, Hitachi, Tokyo, Japan) using ninhydrin for post-column derivatization, following the procedures of Cui et al. [17].

Serial sections (3 to 5 μm) of samples (2 cm^3^) were cut and stained with hematoxylin and eosin to observe the morphology of the muscle tissue. Images were captured by microscopy at 200× magnification. An image analyzer (Image-pro Plus 6.0, Media Cybernetics, Inc., Rockville, MD, USA) was used to score the diameter (μm) and the density (fibers/mm^2^) of the muscle fibers, following the procedures of Cui et al. [17].

### 2.4. Sensory Evaluation

After storage at −20 °C, muscle samples were used for sensory panel testing, following the procedures of Cui et al. [18]. Ten consumers with experience in sensory analysis of poultry meat were invited to evaluate these indicators. The selection criteria were as follows: age between 20 and 50 years, no allergy to chicken, and willing to taste meat from chickens fed experimental diets. Taste samples were placed in a foil-sealed dish and immediately steamed with boiling water for 10 min, until the center temperature of the pectoral muscle reached 80 °C. The chickens for tasting were all numbered, and the sample numbers were hidden. The consumers gargled water between each sample tasting. Sensory evaluation attributes (color and appearance, odor, flavor, tenderness, juiciness, and broth freshness) were blindly rated on a 5-point scale (1 = extremely dissatisfied and 5 = extremely satisfied).

### 2.5. Biochemical Assay of Plasma and Tissue Samples

Tissue homogenates were centrifuged at 12,000× *g* at 4 °C for 10 min, and the supernatants were stored at −80 °C until the biochemical assays. All samples were measured in three copies with appropriate dilution. Glutathione (GSH), oxidized glutathione (GSSG), total superoxide dismutase (T-SOD), lactate dehydrogenase (LDH), calpain (CAPN), calpastatin (CAST), glycogen phosphorylase (GP), glycogen synthase (GS), malate dehydrogenase (MDH), hormone-sensitive lipase (HSL), total triglyceride (TG), total cholesterol (TCH), urea nitrogen (BUN), lactic acid (LD), and uric acid (UA) were measured by a microplate reader (Biomate 5, Thermo Electron Corporation, Rochester, NY, USA). Colorimetric kits (Nanjing Jiancheng Bioengineering Institute, Nanjing, China) were used to measure GSH, GSSG, T-SOD, LDH, TG, TCH, BUN, LD, and UA. CAPN, CAST, GP, GS, MDH, and HSL were determined using chicken ELISA kits (Beijing Equation Biotechnology co., Ltd., Beijing, China). The specific detection methods and result calculations of each index were carried out in accordance with the instructions.

### 2.6. The RNA Extraction and Real-Time Quantitative PCR

The total RNA was isolated and reverse transcribed and put through a real-time quantitative PCR program, as described in detail by Cui et al. [18]. The commercial gene primers were used based on chicken sequences (Sangon Biological Engineering Co., Ltd., Shanghai, China). In this study, we selected *β-actin* as the housekeeping gene for normalization purposes. Primer Premier 6.0 software (Premier Biosoft International, Palo Alto, CA, USA) was used to design specific primers for six genes (Supplementary Table S1), including fatty acid synthase (*FAS*), acetyl-CoA carboxylase (*ACC*), peroxisome proliferators-activated receptors α (*PPAR-α*), and sterol regulatory element binding protein-1c (*SREBP-1c*) genes.

### 2.7. Determination of Cecal Microbiota

The total genomic DNA was extracted, and the product of DNA amplification was amplified by PCR using primers for the V4 domain of the bacterial 16S rRNA gene [19]. The Ion Plus Fragment Library Kit (Thermo Fisher Scientific, Waltham, MA, USA) was used to construct libraries. Based on the IonS5XL sequencing platform of Novogene Bioinformatics Technology Co., LTD. (Beijing, China), a small fragment library was constructed using the single-end sequencing method after Qubit quantification (Qubit 2.0 fluorometer, Life Technology, Carlsbad, CA, USA) and library testing. The clean data were obtained by reading data through cutting and filtering, and the sequences were clustered into operational taxonomic units (OTU) with 97% similarity. Species annotation analysis was performed using OTU sequences and the Silva132 database [20]. According to the species notes, the differences in community structure among different treatments were analyzed by calculating alpha diversity and beta diversity [19].

### 2.8. Statistical Analysis

The effect of dietary treatments was assessed using a one-way ANOVA test and, where appropriate, using the Tukey post-hoc test in version SPSS 17.0 (SPSS Inc., Chicago, IL, USA). Values are expressed as mean ± standard error of the mean (SEM), derived from the root mean square error of the ANOVA. The difference was considered to be statistically significant at *p* < 0.05.

## 3. Results

### 3.1. Growth Performance and Carcass Quality

As shown in Table 2, compared with the CON group, FR significantly reduced the growth performance of Bearded chickens, showing a significant decline with the degree of FR (*p* < 0.05). However, compared with the CON group, there was no significant difference in FCR and slaughtering weight in the 90% FI, 80% FI, and 90% ME groups (*p* > 0.05). Notably, FR had no adverse effects on the carcass quality of Bearded chickens (*p* > 0.05). Except for the 70% ME group, the abdominal fat rate decreased with FR degree compared to the CON group (*p* > 0.05). Therefore, it showed that the yield of the FR group improved, yet the total weight of the chickens was slighter.

### 3.2. Biochemical Assay of Plasma and Tissue Samples

As shown in Table 3, compared with the CON group, only 80% FI showed a significant increase in the content of GSH and UA in plasma (*p* < 0.05) and CAPN activity in the breast muscle (*p* < 0.05). Meanwhile, the content of TG, TCH, BUN, and LDH in plasma and CAST activity in breast muscle of the 80% FI chickens were significantly reduced (*p* < 0.05). Additionally, 70% ME significantly increased the content of UA in plasma (*p* < 0.05); significantly reduced the content of TG, TCH, BUN, and LDH in plasma (*p* < 0.05); and significantly reduced CAST activity in the muscle (*p* < 0.05). Importantly, the content of fat, TG, and TCH, as well as *FAS* activity in the liver, was also significantly reduced in the 80% FI and 70% ME groups (*p* < 0.05).

### 3.3. The mRNA Expression of Key Genes in the Lipid Metabolism of the Liver

As shown in Table 4, compared with the CON group, the mRNA expression of the *FAS*, *ACC*, *PPAR-α*, and *SREBP-1c* of the liver in the 80% FI group significantly decreased (*p* < 0.05). The 90% ME, 80% ME, and 70% ME groups had significantly reduced expression of *FAS* and *ACC* mRNA in the liver (*p* < 0.05). Moreover, the mRNA expression of *PPAR-α* and *SREBP-1c* of the chicken in the 70% ME group was also significantly reduced (*p* < 0.05).

### 3.4. Cecal Microbial Community

#### 3.4.1. Alpha Diversity of Cecum Microbiota

As shown in Table 5, many indices (OTU numbers, Chao1, ACE, Simpson, Shannon, and PD_whole_tree index) reflect the alpha diversity of microbial communities, and an analysis of the OTU numbers and Chao1 showed that the OTU numbers and Chao1 index of 80% FI were significantly higher than that of 70% ME (*p* < 0.05).

#### 3.4.2. Beta Diversity of Cecum Microbiota

**Figure 1:**
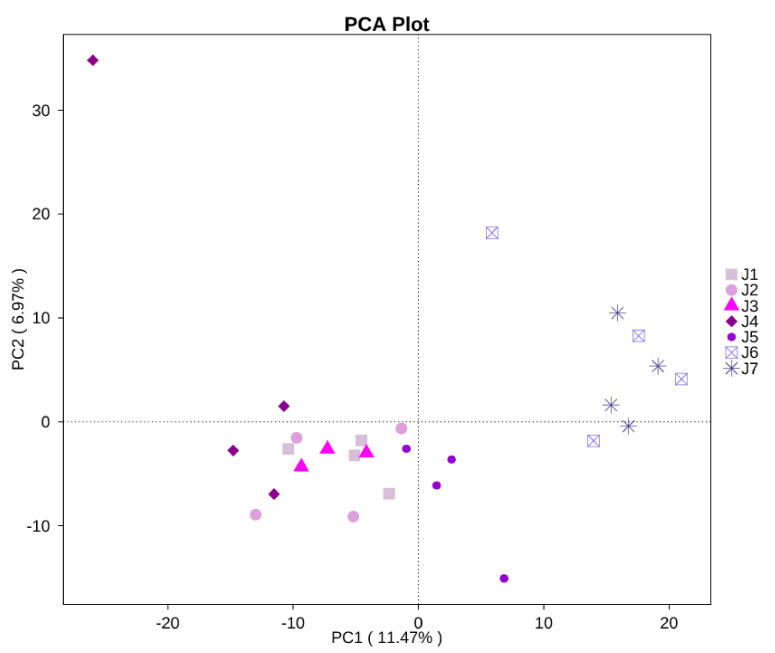
Principal component analysis (PCA) plot of the cecal microbiota. J1 = CON, fed with the basal diet ad libitum; J2 = 90% FI, fed with 90% feed intake of the CON; J3= 80% FI, fed with 80% feed intake of the CON; J4 = 70% FI, fed with 70% feed intake of the CON; J5 = 90% ME, fed with 90% metabolizable energy of the CON; J6 = 80% ME, fed with 80% metabolizable energy of the CON; J7 = 70% ME, fed with 70% metabolizable energy of the CON.

As shown in Figure 1, the cecal microflora of CON and qualitative FR groups showed significant differentiation, while the separation between CON and quantitative FR groups was almost invisible. The microbial community composition of the CON group was more similar to that of the 80% FI group than that of the 70% ME group, and the degree of dispersion among the group 80% FI samples was less than that among the group 70% ME samples.

**Figure 2:**
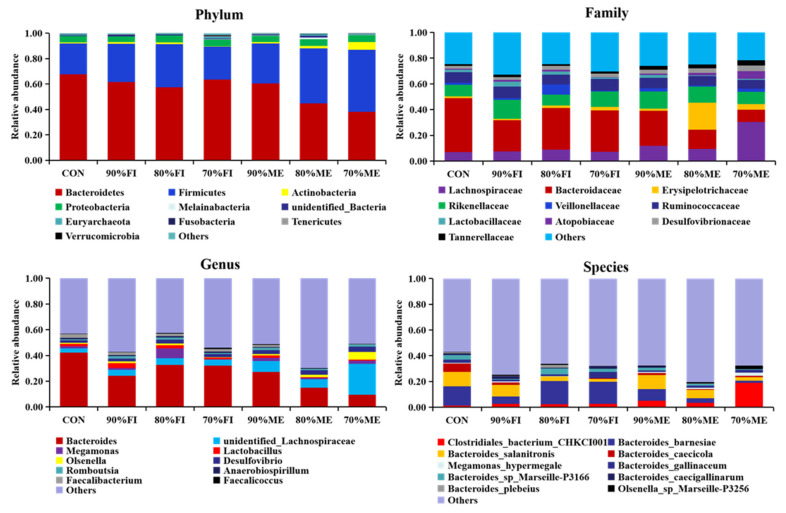
Composition and distribution of the microbiota at the phylum, family, genus, and species level. CON = fed with the basal diet ad libitum; 90% FI = fed with 90% feed intake of the CON; 80% FI = fed with 80% feed intake of the CON; 70% FI = fed with 70% feed intake of the CON; 90% ME = fed with 90% metabolizable energy of the CON; 80% ME = fed with 80% metabolizable energy of the CON; 70% ME = fed with 70% metabolizable energy of the CON.

As shown in Figure 2, a total of 10 phylum, 10 family, 10 genus and 10 species were found in all samples. The cecal flora of each group were mainly composed of Bacteroidetes, Firmicutes, Actinobacteria and Proteobacteria, and the dominant bacteria were Firtinobacteria and Bacteroidetes. Compared to the CON group, the proportion of Bacteroidetes in FR groups decreased, while the proportion of Firmicutes and Actinobacteria increased. The effect of quantitative FR on microflora structure was less than that of qualitative FR. The lower the dietary energy level, the higher the proportion of Firmicutes and Actinobacteria in the cecum of Bearded chickens.

**Figure 3:**
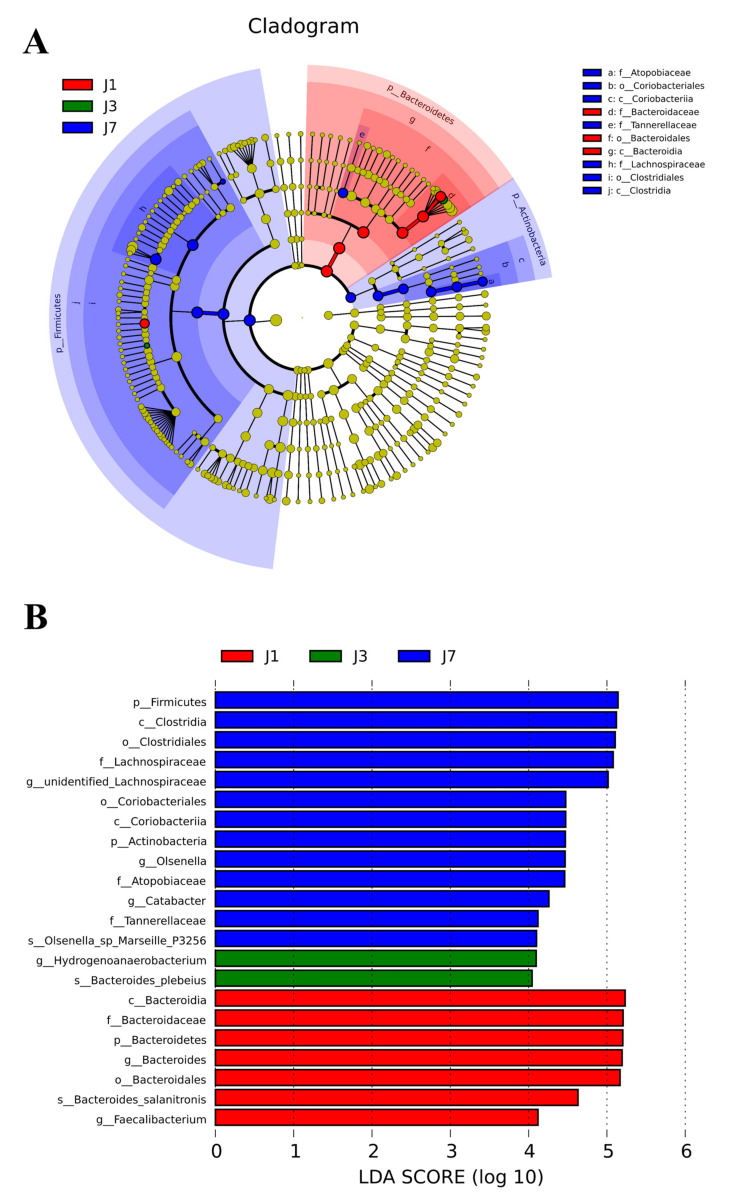
Linear discriminant analysis (LDA) combined with effect size (LEfSe) identified the most diverse groups in chicken cecal flora. (**A**) Taxonomic lineage plots were obtained from LEfSe analysis of 16S rRNA sequencing. Biomarker groups are represented by colored circles and shaded areas. The diameter of each circle is related to the abundance of taxa in the community. (**B**) Only the taxa met the significance threshold >4 for the linear discriminant analysis. J1 = CON, fed with the basal diet ad libitum; J3 = 80% FI, fed with 80% feed intake of the CON; J7 = 70% ME, fed with 70% metabolizable energy of the CON.

As shown in Figure 3, Linear discriminant analysis effect size (LEfSe) analysis of the cecum bacterial community showed that there were compositional differences between the FR group and the CON group. An increase in the abundance of *Hydrogenoanaerobacterium* (genus) and *Bacteroides plebeius* (species) was observed in the J3 (80% FI) group. An increase in the abundance of Actinobacteria (phylum), Coriobacteriia (class), Coriobacteriales (order), Atopobiaceae (family), *Olsenella* (genus), *Olsenella_sp_Marseille_P3256* (species) and an enrichment in Firmicutes (phylum), Clostridia (class), Clostridiales (order), *Catabacter* (genus), and *unidentified_Lachnospiraceae* (genus) were observed in the J7 (70% ME) group.

### 3.5. Meat Quality and Sensory Evaluation

As shown in Table 6, compared with the CON group, the shear force of the breast muscle in the 80% FI and 70% ME groups decreased by 19.12% (*p* > 0.05) and 17.93% (*p* > 0.05), respectively. The L * value of the breast muscle at 45 min after slaughter in the 90% FI group was significantly reduced (*p* < 0.05), and the a * value at 24 h significantly improved (*p* < 0.05). The L * value (45 min and 24 h) significantly decreased, and the a* value significantly increased in the 80% FI group (*p* < 0.05). There were no adverse effects on drip loss, pH, tissue characteristics of muscle fiber, IMF, and IMP in the breast muscle of Bearded chickens in the FR groups (*p* > 0.05).

Sensory evaluation of the meat quality is summarized in Table 7. Only the color and appearance of the breast muscle in the 70% FI group were significantly reduced (*p* < 0.05). FR has no negative effects on the sensory scores of Bearded chicken breast muscle (*p* > 0.05). Notably, compared to the CON group, the color and appearance, odor, tenderness, juiciness, and broth freshness of the 80% FI group increased by 6.70%, 3.09%, 14.20%, 2.93%, and 2.93%, respectively (*p* > 0.05).

### 3.6. Amino Acids of the Breast Muscle

As shown in Table 8, the concentration of free amino acids including threonine, lysine, aspartic acid, glutamic acid, proline, and arginine was higher in the 80% FI group than that in the CON group (*p* < 0.05). The groups 90% ME, 80% ME, and 70% ME had significantly decreased contents of valine, isoleucine, leucine, phenylalanine, lysine, alanine, tyrosine, and proline in the breast muscle (*p* < 0.05) but there were no significant effects on the contents of aspartic acid, glutamic acid, glycine, and arginine (*p* > 0.05).

## 4. Discussion

The main cost for poultry and other intensive livestock producers is the cost of feed, which has become an important issue, as the price of feed raw materials continues to rise [21]. Massuquetto et al. [22] reported that feed intake reduction can result in lower performance and lower carcass and cuts yield in broiler chickens. Chen et al. [2] found that the energy restriction broiler chickens had significantly lower ADG and relative weight gain (RWG) at the early stage of experiment (18–39 d) compared to the ad libitum group, while at the latter stage of experiment (40–48 d), the RWG of the energy restriction broiler chickens was higher than that of the ad libitum group. In the present study, the growth performance of chickens showed a significant downward trend with the increase in FR, which was consistent with previous findings [2,7,12,22].

Abdominal and subcutaneous fat are regarded as the main sources of waste in the slaughterhouse. The use of FR to reduce fat deposition has received considerable attention. Chen et al. [2] have reported that the abdominal fat percentage and subcutaneous fat thickness in the 30% energy-restricted Arbor Acre broiler group were 35% and 75.57% of those in the ad libitum group, respectively. Additionally, there was no significant difference in leg muscle ratio and breast muscle ratio between the ad libitum and FR broiler chickens [2,23,24]. In our study, FR had a tendency to increase the muscle percentage and decrease the abdominal fat percentage of Bearded chickens, caused by higher physical activity in search of feed. These results indicated that FR of Bearded chickens at the finisher phase improved the carcass performance to a certain extent, basically consistent with the reports of Chen et al. [2], Englmaierová et al. [23], and Jahanpour et al. [24]. Other studies have also shown that restricted feeding can improve the economic performance of chickens at different growth stages [25,26].

Furthermore, 16S rRNA sequencing indicated that chickens in the 70% ME group have an increase in the abundance of Firmicutes and Actinobacteria, which were significantly negatively correlated with most of the lipogenesis indicators [14]. However, our study found a decrease in the proportion of Bacteroidetes in the FR group, which was inconsistent with Xiang et al. [15], who found that the decrease of Bacteroidetes and the increase in Firmicutes were correlated with the accumulation of abdominal fat deposition in genetically selected chickens. In this study, it was found for the first time that the effect of quantitative FR on microflora structure was less than that of qualitative FR. Fortunately, an increase in the abundance of the beneficial bacteria *Hydrogenoanaerobacterium* (genus) [19,27] and *Bacteroides plebeius* (species) [28] was observed in the 80% FI group. Moreover, chickens in the 70% ME group had an increase in the abundance of *Olsenella* and *Lachnospiraceae*, which were significantly negatively correlated with abdominal fat deposition [14]. Additionally, 80% FI and 70% ME also significantly reduced the content of TG and TCH in plasma, and the content of fat, TG, and TCH, as well as the relative expression of *FAS*, *ACC*, *PPAR-α*, and *SREBP-1c* in the liver, which was partially in agreement with previous studies [29]. It can be concluded that FR of Bearded chickens regulates lipid metabolism by changing the structure of the cecal content of the flora, thereby improving carcass quality. However, the effect of FR on plasma TG was different from the result of Jahanpour et al. [30], and it is hypothesized that this might be related to the broiler breed and restricted feeding stage. Chen et al. [2] have reported that FR in broiler chickens produces the effect of alleviating oxidative stress, which also corresponds with our study that 80% FI significantly decreased lipid peroxidation damage by increasing the content of GSH in plasma. This also shows that FR of Bearded chickens can significantly improve the body’s blood–lipid metabolism, which is more conducive to the health of the chickens.

FR has been adopted to avoid a rapid growth rate, which is considered responsible for poor meat quality [31,32]. For example, Kawasaki et al. [32] reported that rapid growth in broiler chickens might be a cause of remarkably hardened breast. Physical indices, such as shearing force, drip loss, flesh color and pH, reflect the edible quality and economic value of muscle [17,18,33]. It was reported that the shear force was reduced as the fat content increased in muscle [33]. The current study observed a decrease in the shear force of the breast muscle by 19.12% and 17.93% in the 80% FI and 70% ME groups, respectively. Consistently, the IMF in the breast muscle of broiler chickens in 80% FI and 70% ME groups increased by 8.92% and 8.01%, respectively. Histological characteristics of muscle fibers were not only used to evaluate the tenderness of the meat but were also closely related to physical characteristics. Early FR of Ross 308 chickens may not affect the number of muscle fibers per 1 mm^2^ diameter, but the fiber cross-sectional area can be enlarged in restricted chickens and affected by the FR intensity [34]. Amazingly, the study by Englmaierová et al. [23] on Hubbard JA757 cockerels found that the number of muscle fibers increased, and the area and diameter decreased with increasing levels of restriction. There were no negative effects on the tissue characteristics of the muscle fiber by FR in our study. This further indicates that the muscle fiber quantity of chickens is determined before hatching, and the pre-market feeding restriction has little effect on it. The calpain system (calpains, CAPN; calpastatin, CAST) plays an important role in postmortem tenderization of skeletal muscle due to its involvement in the degradation of important myofibrillar and associated proteins, as well as in cytoskeletal remodeling and regulation of muscle growth [35,36,37,38]. In the present study, the activity of CAPN and CAST were significantly affected in the 80% FI group, which further confirmed that this group could improve meat quality by reducing muscle shear force and drip loss. Nevertheless, Lippens et al. [39] showed no effect of FR of chickens on the pH and color of breast muscles. Additionally, our data also showed that 80% FI tended to decrease the L * value and increase the a * value, which both benefit the meat quality. However, the differences in the results of different studies are largely related to the species studied.

The flavor of meat is closely related to its content of IMP and the compositions of amino acids, especially the aspartic acid, glutamic acid, glycine, and arginine [17,18]. Surprisingly, in our study, the breast muscles of FR chickens showed a trend for higher IMP. The concentrations of threonine, lysine, aspartic acid, glutamic acid, proline and arginine was higher in the 80% FI group than in the CON group. Importantly, flavor identification results showed that the 80% FI group scored higher. Accordingly, it was further confirmed that chickens in the 80% FI group had better flavor and were more popular with consumers, which was consistent with the above various indicators.

## 5. Conclusions

In conclusion, the results obtained clearly indicate that FR increased meat yield, but the total weight of the Bearded chickens was slighter compared to that the CON group; 80% FI and 70% ME could improve lipid metabolism by changing the structure of the cecal microbial community, and the meat quality and flavor of Bearded chickens in the 80%FI group improved in terms of promoted meat color index, flavor substances, and calproteinase system. Considering various indicators of tasting, the 80% FI is the most suitable for Bearded chickens before marketing.

## Figures and Tables

**Table 1:** Dietary composition and nutrient level of Bearded chickens in each treatment group at the finisher phase (DM basis, %).

Ingredients		Quantitative FR			Qualitative FR		
	CON	90% FI	80% FI	70% FI	90% ME	80% ME	70% ME
Corn	72.50	72.50	72.50	72.50	58.40	44.30	30.30
Wheat bran	4.80	4.80	4.80	4.80	4.60	4.40	4.20
Unite bran	0.00	0.00	0.00	0.00	12.81	25.60	38.20
Soybean meal	14.90	14.90	14.90	14.90	15.40	15.90	16.40
Soybean oil	3.00	3.00	3.00	3.00	3.00	3.00	3.00
L-lysine HCl	0.29	0.29	0.29	0.29	0.27	0.25	0.23
DL-Methionine	0.21	0.21	0.21	0.21	0.25	0.28	0.32
L-Threonine	0.06	0.06	0.06	0.06	0.06	0.06	0.06
Isoleucine	0.07	0.07	0.07	0.07	0.06	0.06	0.05
Limestone	1.20	1.20	1.20	1.20	1.08	0.97	0.86
Dicalcium phosphate	1.51	1.51	1.51	1.51	1.5	1.48	1.46
Salt	0.30	0.30	0.30	0.30	0.30	0.30	0.30
Zeolite	0.16	0.16	0.16	0.16	1.27	2.40	3.62
Vitamin–mineral premix ^1^	1.00	1.00	1.00	1.00	1.00	1.00	1.00
Total	100.00	100.00	100.00	100.00	100.00	100.00	100.00
Calculated nutrient composition ^2^							
Metabolizable energy (kcal·kg^−1^)	3047	3047	3047	3047	2742	2438	2133
Crude protein	14.00	14.00	14.00	14.00	14.00	14.00	14.00
Crude fiber	2.08	2.08	2.08	2.08	5.26	8.43	11.55
Ether extract	5.85	5.85	5.85	5.85	6.02	6.19	6.35
Lysine	0.85	0.85	0.85	0.85	0.85	0.85	0.85
Methionine	0.38	0.38	0.38	0.38	0.46	0.49	0.53
Methionine + cysteine	0.65	0.65	0.65	0.65	0.65	0.65	0.65
Threonine	0.58	0.58	0.58	0.58	0.58	0.58	0.58
Tryptophan	0.15	0.15	0.15	0.15	0.16	0.17	0.17
Isoleucine	0.57	0.57	0.57	0.57	0.57	0.57	0.57
Calcium	0.85	0.85	0.85	0.85	0.85	0.85	0.85
Non-phytate phosphorus	0.36	0.36	0.36	0.36	0.36	0.36	0.36

CON = fed with the basal diet ad libitum; 90% FI = fed with 90% feed intake of the CON; 80% FI = fed with 80% feed intake of the CON; 70% FI = fed with 70% feed intake of the CON; 90% ME = fed with 90% metabolizable energy of the CON; 80% ME = fed with 80% metabolizable energy of the CON; 70% ME = fed with 70% metabolizable energy of the CON. ^1^ The premix provided the following per kg of the diet: chicken multi-dimensional C428 200 mg, baking soda 1500 mg, poultry mine 800 mg, zeolite powder 4500 mg, choline chloride 1500 mg. ^2^ Except where indicated, nutrient levels are all calculated values.

**Table 2:** Feed restriction (FR) increased meat yield, but the total weight of the Bearded chickens was slighter.

		Quantitative FR			Qualitative FR				
Parameter ^1^	CON	90% FI	80% FI	70% FI	90% ME	80% ME	70% ME	SEM	*p*-Value
Growth performance									
150 d BW, g	1414.88	1414.75	1413.50	1413.50	1413.75	1415.00	1415.50	0.26	0.231
190 d BW, g	1764.44 ^a^	1706.46 ^b^	1645.90 ^c^	1579.03 ^d^	1715.63 ^b^	1580.49 ^d^	1512.50 ^e^	12.29	<0.001
ADFI, g	69.84 ^a^	62.81 ^c^	55.85 ^d^	48.87 ^e^	69.23 ^b^	69.33 ^b^	69.21 ^b^	1.04	<0.001
ADG, g	8.74 ^a^	7.29 ^b^	5.81 ^c^	4.14 ^d^	7.55 ^b^	4.14 ^d^	2.43 ^e^	0.31	<0.001
FCR	8.09 ^d^	8.68 ^d^	9.90 ^cd^	12.27 ^c^	9.35 ^cd^	17.86 ^b^	25.84 ^a^	0.84	<0.001
Carcass quality									
Slaughtering weight, g	1577.00 ^a^	1500.00 ^ab^	1479.67 ^abc^	1440.33 ^bc^	1558.00 ^a^	1399.00 ^cd^	1316.00 ^d^	21.45	0.001
Dressing percentage	88.49	88.38	89.90	89.65	89.44	89.97	88.19	0.40	0.836
Breast muscle yield, %	12.46	15.12	15.41	17.22	16.58	15.49	15.46	0.71	0.702
Thigh muscle yield, %	16.90	23.24	20.53	23.01	21.60	21.67	20.80	1.06	0.764
Abdominal fat, %	10.80 ^a^	10.57 ^a^	9.08 ^ab^	8.91 ^ab^	8.89 ^ab^	7.89 ^ab^	6.64 ^b^	0.44	0.206

SEM: standard error of mean, *n* = 8. CON= fed with the basal diet ad libitum; 90% FI = fed with 90% feed intake of the CON; 80% FI= fed with 80% feed intake of the CON; 70% FI = fed with 70% feed intake of the CON; 90% ME = fed with 90% metabolizable energy of the CON; 80% ME = fed with 80% metabolizable energy of the CON; 70% ME = fed with 70% metabolizable energy of the CON. ^1^ BW = body weight; ADFI = average daily feed intake; ADG = average daily gain; FCR = feed conversion rate. In the same row, values with different same letter superscripts (^a–e^) represent significant difference (*p* < 0.05), while with the same or no letter superscripts represent no significant difference (*p* > 0.05).

**Table 3:** Effects of feed restriction (FR) on plasma, breast muscle, and liver biochemical indices of Bearded chickens.

		Quantitative FR			Qualitative FR				
Parameter ^1^	CON	90% FI	80% FI	70% FI	90% ME	80% ME	70% ME	SEM	*p*-Value
Plasma									
GSH, mg/L	16.23 ^b^	18.67 ^ab^	22.58 ^a^	17.56 ^b^	14.76 ^b^	18.31 ^ab^	17.81 ^b^	0.67	0.042
GSSG, μmol/L	113.30	103.44	116.44	117.57	115.55	119.42	109.74	1.89	0.456
GSH/GSSG	0.16 ^ab^	0.19 ^a^	0.19 ^a^	0.16 ^ab^	0.13 ^b^	0.12 ^b^	0.15 ^ab^	0.01	0.028
T-SOD, U/mL	585.94	571.14	567.10	604.55	579.53	575.08	596.95	13.66	0.991
TG, mmol/L	2.64 ^a^	2.10 ^ab^	1.05 ^c^	1.75 ^bc^	2.03 ^a^	2.34 ^a^	1.51 ^bc^	0.14	0.021
TCH, mmol/L	8.68 ^a^	8.61 ^a^	6.42 ^b^	7.95 ^ab^	8.94 ^a^	8.08 ^ab^	7.00 ^b^	0.24	0.026
BUN, mmol/L	1.21 ^ab^	1.01 ^abc^	0.78 ^c^	0.93 ^bc^	1.28 ^a^	0.97 ^abc^	0.88 ^c^	0.05	0.032
UA, mg/L	31.41 ^c^	45.24 ^ab^	53.39 ^a^	46.38 ^ab^	41.33 ^bc^	42.88 ^abc^	46.93 ^ab^	1.73	0.032
LDH, U/L	2692.62 ^a^	2368.85 ^ab^	1765.71 ^c^	2353.63 ^ab^	2500.00 ^ab^	2304.19 ^b^	1832.88 ^c^	64.13	<0.001
Breast muscle									
CAPN, ng/g prot	21.31 ^b^	23.91 ^ab^	27.81 ^a^	25.65 ^ab^	21.86 ^b^	25.37 ^ab^	26.14 ^ab^	0.69	0.155
CAST, ng/g prot	7.94 ^a^	5.40 ^bc^	4.86 ^c^	5.77 ^bc^	7.05 ^ab^	6.23 ^abc^	5.55 ^bc^	0.27	0.026
GP, ng/g prot	28.82	26.63	29.44	26.28	30.76	32.81	32.10	0.92	0.406
GS, nmol/g prot	0.80	0.78	0.88	0.72	0.78	0.99	0.86	0.04	0.639
LD, mmol/g prot	1.18	1.04	1.03	1.13	1.08	1.06	1.09	0.02	0.708
Liver									
Fat content, g/kg	19.63 ^a^	20.84 ^a^	15.12 ^b^	15.78 ^b^	16.09 ^b^	15.86 ^b^	13.15 ^b^	0.54	<0.001
FAS, nmol/g prot	0.73 ^a^	0.72 ^a^	0.61 ^b^	0.66 ^ab^	0.67 ^ab^	0.65 ^ab^	0.59 ^b^	0.01	0.046
MDH, ng/g prot	6.42	6.25	5.91	6.01	5.71	6.25	6.05	0.12	0.785
HSL, μg/g prot	2.96	3.21	3.30	3.13	2.95	2.95	3.06	0.05	0.459
TG, mmol/g prot	0.12 ^ab^	0.14 ^a^	0.08 ^d^	0.10 ^bcd^	0.10 ^bc^	0.08 ^cd^	0.08 ^d^	0.00	<0.001
TCH, mmol/g prot	0.18 ^a^	0.11 ^b^	0.10 ^b^	0.11 ^b^	0.11 ^b^	0.11 ^b^	0.09 ^b^	0.01	0.018

CON = fed with the basal diet ad libitum; 90% FI = fed with 90% feed intake of the CON; 80% FI = fed with 80% feed intake of the CON; 70% FI = fed with 70% feed intake of the CON; 90% ME = fed with 90% metabolizable energy of the CON; 80% ME = fed with 80% metabolizable energy of the CON; 70% ME= fed with 70% metabolizable energy of the CON. ^1^ GSH = reduced glutathione; GSSG = oxidized glutathione; T-SOD = total superoxide dismutase; LDH = lactate dehydrogenase; CAPN = calpain; CAST = calpastatin; GP = glycogen phosphorylase; GS = glycogen synthase; MDH = malate dehydrogenase; HSL = hormone-sensitive lipase; TG = total triglyceride; TCH = total cholesterol; BUN = urea nitrogen; LD = lactic acid; UA = uric acid. In the same row, values with different same-letter superscripts (^a–d^) represent significant difference (*p* < 0.05), while those with the same or no letter superscripts represent no significant difference (*p* > 0.05).

**Table 4:** Effects of feed restriction (FR) on mRNA expression of key genes in lipid metabolism in the livers of Bearded chickens.

		Quantitative FR			Qualitative FR				
Genes ^1^	CON	90% FI	80% FI	70% FI	90% ME	80% ME	70% ME	SEM	*p*-Value
*FAS*	1.21 ^a^	1.06 ^a^	0.26 ^c^	0.88 ^a^	0.56 ^bc^	0.58 ^b^	0.31 ^bc^	0.07	<0.001
*ACC*	1.72 ^a^	0.72 ^b^	0.34 ^b^	1.04 ^b^	0.52 ^b^	0.44 ^b^	0.48 ^b^	0.12	0.004
*PPAR-* *α*	1.03 ^b^	0.44 ^c^	0.29 ^c^	1.08 ^b^	1.64 ^a^	1.12 ^b^	0.29 ^c^	0.11	<0.001
*SREBP-1c*	1.24 ^a^	1.11 ^a^	0.52 ^bc^	1.21 ^a^	0.88 ^abc^	1.03 ^ab^	0.34 ^c^	0.09	0.016

CON = fed with the basal diet ad libitum; 90% FI = fed with 90% feed intake of the CON; 80% FI = fed with 80% feed intake of the CON; 70% FI = fed with 70% feed intake of the CON; 90% ME = fed with 90% metabolizable energy of the CON; 80% ME = fed with 80% metabolizable energy of the CON; 70% ME = fed with 70% metabolizable energy of the CON. ^1^  *FAS* = fatty acid synthase; *ACC* = acetyl-CoA synthetase carboxylase; *PPAR-α* = peroxisome proliferators-activated receptors; *SREBP-1c* = sterol regulatory element binding protein-1c. In the same row, values with different same-letter superscripts (^a–c^) represent significant difference (*p* < 0.05), while those with the same- or no-letter superscripts represent no significant difference (*p* > 0.05).

**Table 5:** Alpha diversity analysis of the microbiota from the cecum of Bearded chickens based on 97% sequence similarity.

		Quantitative FR			Qualitative FR				
Items ^1^	CON	90% FI	80% FI	70% FI	90% ME	80% ME	70% ME	SEM	*p*-Value
OTU numbers	514.50 ^a^	545.75 ^a^	508.33 ^a^	562.25 ^a^	553.25 ^a^	514.50 ^ab^	467.00 ^b^	7.68	0.002
Coverage, %	99.78	99.80	99.80	99.83	99.78	99.75	99.83	0.01	0.215
Community richness									
Chao1	574.42 ^a^	576.83 ^a^	568.12 ^a^	594.22 ^a^	582.68 ^a^	557.23 ^ab^	501.37 ^b^	7.67	0.002
ACE	574.36 ^ab^	589.93 ^a^	560.40 ^ab^	604.18 ^a^	612.09 ^a^	572.34 ^ab^	515.14 ^b^	7.74	0.004
Community diversity									
Shannon	6.02 ^ab^	6.69 ^a^	6.08 ^ab^	6.43 ^ab^	6.44 ^ab^	5.89 ^b^	5.88 ^b^	0.08	0.008
Simpson	0.95	0.98	0.95	0.96	0.97	0.95	0.96	0.00	0.227
PD_whole_tree index	42.75	41.95	40.49	44.83	41.66	45.16	38.95	0.73	0.220

CON = fed with the basal diet ad libitum; 90% FI = fed with 90% feed intake of the CON; 80% FI = fed with 80% feed intake of the CON; 70% FI = fed with 70% feed intake of the CON; 90% ME = fed with 90% metabolizable energy of the CON; 80% ME = fed with 80% metabolizable energy of the CON; 70% ME = fed with 70% metabolizable energy of the CON. ^1^ OTU = operational taxonomic units; ACE = abundance-based coverage estimator. The variant letter in the same row indicates significant difference when *p* < 0.05.

**Table 6:** Feed restriction (FR)-improved meat color index of Bearded chickens.

		Quantitative FR			Qualitative FR				
Parameter ^1^	CON	90% FI	80% FI	70% FI	90% ME	80% ME	70% ME	SEM	*p*-Value
Shear force, N	31.06	27.60	25.12	28.14	27.53	28.03	25.49	0.71	0.388
Drip loss, %	2.12	1.93	1.80	1.92	1.95	1.97	1.88	0.05	0.817
pH 45 min	6.07	6.20	6.23	6.16	6.09	6.21	6.11	0.03	0.838
pH 24 h	5.63	5.65	5.69	5.64	5.65	5.68	5.68	0.01	0.508
L* value 45 min	58.76 ^ab^	56.62 ^c^	56.41 ^c^	57.46 ^bc^	58.85 ^ab^	57.62 ^bc^	60.01 ^a^	0.26	<0.001
a* value 45 min	10.37 ^b^	10.69 ^b^	11.78 ^a^	11.10 ^ab^	10.60 ^b^	10.53 ^b^	10.32 ^b^	0.12	0.005
b* value 45 min	12.52 ^ab^	12.70 ^a^	12.65 ^ab^	13.79 ^a^	12.96 ^a^	12.45 ^ab^	10.61 ^b^	0.27	0.167
L* value 24 h	60.91 ^a^	59.54 ^ab^	58.89 ^b^	59.83 ^ab^	61.01 ^a^	60.22 ^ab^	61.10 ^a^	0.24	0.109
a* value 24 h	10.15 ^c^	11.67 ^a^	11.24 ^ab^	10.34 ^bc^	10.76 ^abc^	10.98 ^abc^	10.67 ^bc^	0.38	0.032
b* value 24 h	14.12 ^a^	14.08 ^a^	14.40 ^a^	14.35 ^a^	14.02 ^ab^	13.20 ^ab^	11.93 ^b^	0.29	0.206
IMF, g/kg	4.37	4.56	4.76	4.77	4.43	4.56	4.72	0.11	0.954
IMP, g/kg	1.80	1.92	2.10	1.79	2.05	2.06	2.12	0.05	0.394
Histological character of muscle fiber									
Fiber diameters, μm	55.08	55.06	53.25	57.47	54.56	54.96	53.30	0.76	0.803
Fiber density, fibers/mm^2^	235.15	231.67	250.94	228.64	242.63	247.05	256.13	7.84	0.965

CON = fed with the basal diet ad libitum; 90% FI = fed with 90% feed intake of the CON; 80% FI = fed with 80% feed intake of the CON; 70% FI= fed with 70% feed intake of the CON; 90% ME = fed with 90% metabolizable energy of the CON; 80% ME = fed with 80% metabolizable energy of the CON; 70% ME = fed with 70% metabolizable energy of the CON. The ^1^ a * = redness; the b * = yellowness; the L * = lightness; IMF = intramuscular fat; IMP = inosine-5′-monphosphate. In the same row, values with different same-letter superscripts (^a–c^) represent significant difference (*p* < 0.05), while those with the same- or no-letter superscripts represent no significant difference (*p* > 0.05).

**Table 7:** Feed restriction (FR)-improved sensory indices of Bearded chickens.

		Quantitative FR			Qualitative FR				
Parameter	CON	90% FI	80% FI	70% FI	90% ME	80% ME	70% ME	SEM	*p*-Value
Color and appearance	3.88 ^a^	4.00 ^a^	4.14 ^a^	3.25 ^b^	3.75 ^ab^	4.00 ^a^	4.00 ^a^	0.09	0.099
Odor	3.88	3.88	4.00	3.25	3.50	3.57	3.75	0.10	0.474
Flavor	4.00	3.75	3.86	3.75	3.38	3.57	3.63	0.10	0.729
Tenderness	3.38	3.25	3.86	3.25	3.13	3.14	3.38	0.11	0.709
Juiciness	3.75	3.25	3.86	3.63	3.38	3.29	3.38	0.10	0.650
Broth freshness	3.75	3.75	3.86	3.38	3.63	3.71	3.88	0.10	0.889

CON = fed with the basal diet ad libitum; 90% FI = fed with 90% feed intake of the CON; 80% FI = fed with 80% feed intake of the CON; 70% FI = fed with 70% feed intake of the CON; 90% ME = fed with 90% metabolizable energy of the CON; 80% ME = fed with 80% metabolizable energy of the CON; 70% ME = fed with 70% metabolizable energy of the CON. In the same row, values with different same-letter superscripts (^a–b^) represent significant difference (*p* < 0.05), while those with the same- or no-letter superscripts represent no significant difference (*p* > 0.05).

**Table 8:** Effects of feed restriction (FR) on the contents of free amino acid in the muscle of Bearded chickens (g/kg).

		Quantitative FR			Qualitative FR				
Parameter	CON	90% FI	80% FI	70% FI	90% ME	80% ME	70% ME	SEM	*p*-Value
Threonine	1.33 ^c^	2.07 ^bc^	3.46 ^a^	2.77 ^ab^	2.89 ^ab^	2.19 ^bc^	2.25 ^b^	0.15	0.002
Valine	1.80 ^a^	1.74 ^a^	2.14 ^a^	1.78 ^a^	0.91 ^b^	1.00 ^b^	1.03 ^b^	0.09	<0.001
Isoleucine	1.15 ^b^	1.57 ^a^	1.25 ^b^	0.94 ^b^	0.54 ^c^	0.55 ^c^	0.54 ^c^	0.07	<0.001
Leucine	3.45 ^a^	4.03 ^a^	3.80 ^a^	3.28 ^a^	1.50 ^b^	1.29 ^b^	1.18 ^b^	0.20	<0.001
Phenylalanine	2.44 ^a^	2.81 ^a^	2.58 ^a^	2.33 ^a^	0.87 ^b^	0.79 ^b^	0.74 ^b^	0.14	<0.001
Lysine	1.78 ^b^	1.95 ^b^	2.67 ^a^	2.04 ^b^	1.06 ^c^	1.16 ^c^	1.14 ^c^	0.11	<0.001
Aspartic acid	0.21 ^b^	0.24 ^b^	0.31 ^a^	0.30 ^ab^	0.25 ^ab^	0.27 ^ab^	0.28 ^ab^	0.01	0.357
Glutamic acid	3.83 ^b^	3.92 ^b^	6.24 ^a^	6.02 ^a^	4.26 ^b^	4.03 ^b^	4.25 ^b^	0.23	0.006
Glycine	1.87	1.82	1.89	1.97	1.40	1.54	1.40	0.09	0.352
Alanine	4.08 ^a^	4.27 ^a^	4.61 ^a^	3.96 ^a^	2.50 ^b^	2.87 ^b^	2.66 ^b^	0.18	<0.001
Proline	1.27 ^bc^	1.59 ^ab^	1.63 ^a^	1.15 ^cd^	0.85 ^d^	0.93 ^cd^	0.93 ^d^	0.06	<0.001
Arginine	1.12 ^bcd^	1.58 ^abc^	1.75 ^a^	1.19 ^cd^	0.86 ^d^	0.93 ^d^	0.93 ^d^	0.07	0.002
Serine	3.50 ^a^	3.45 ^a^	3.39 ^a^	2.94 ^ab^	2.39 ^b^	2.61 ^ab^	2.84 ^ab^	0.13	0.131
Tyrosine	1.99 ^a^	2.46 ^a^	2.47 ^a^	1.90 ^a^	1.12 ^b^	1.06 ^b^	1.01 ^b^	0.11	<0.001

CON = fed with the basal diet ad libitum; 90% FI = fed with 90% feed intake of the CON; 80% FI = fed with 80% feed intake of the CON; 70% FI = fed with 70% feed intake of the CON; 90% ME = fed with 90% metabolizable energy of the CON; 80% ME = fed with 80% metabolizable energy of the CON; 70% ME = fed with 70% metabolizable energy of the CON. In the same row, values with different same-letter superscripts (^a–d^) represent significant difference (*p* < 0.05), while those with the same- or no-letter superscripts represent no significant difference (*p* > 0.05).

## Data Availability

The data presented in this study are available on request from the corresponding author.

## Supplementary Materials

The following supporting information can be downloaded at: https://www.mdpi.com/article/10.3390/ani12080970/s1, Table S1: Primers for real-time quantitative PCR.

## References

[1] Zheng X.T., Zhang B., Zhang Y.W., Zhong H.A., Nie R.X., Li J.Y., Zhang H., Wu C.X. (2020). Transcriptome analysis of feather follicles reveals candidate genes and pathways associated with pheomelanin pigmentation in chickens. Sci. Rep..

[2] Chen W., Guo Y.M., Huang Y.Q., Shi Y.H., Zhang C.X., Wang J.W. (2012). Effect of energy restriction on growth, slaughter performance, serum biochemical parameters and Lpin2/WDTC1/mRNA expression of broilers in the later phase. J. Poult. Sci..

[3] Mohammadalipour R., Rahmani H.R., Jahanian R., Riasi A., Mohammadalipour M., Nili N. (2017). Effect of early feed restriction on physiological responses, performance and ascites incidence in broiler chickens raised in normal or cold environment. Animal.

[4] Arrazola A., Mosco E., Widowski T.M., Guerin M.T., Kiarie E.G., Torrey S. (2019). The effect of alternative feeding strategies for broiler breeder pullets: 1. Welfare and performance during rearing. Poult. Sci..

[5] Bordin T., Pilotto F., Pesenatto D., Mendonça B.S.D., Daroit L., Rodrigues L.B., Santos E.D.D., Dickel E.L. (2021). Performance of broiler chicken submitted to a quantitative feed restriction program. Trop. Anim. Health Prod..

[6] Sahraei M. (2012). Feed restriction in broiler chickens production: Are view. Biotechnol. Anim. Husb..

[7] Omosebi D.J., Adeyemi O.A., Sogunle M.O., Idowu O.M.O., Njoku C.P. (2014). Effects of duration and level of feed restriction on performance and meat quality of broiler chickens. Arch. Zootec..

[8] Połtowicz K., Nowak J., Wojtysiak D. (2015). Effect of feed restriction on performance, carcass composition and physicochemical properties of the m. pectoralis superficialis of broiler chickens. Ann. Anim. Sci..

[9] Londok J.J.M.R., Rompis J.E.G. (2020). The effect of early feed restriction on the commercial pieces of two broiler chicken strains. IOP Conf. Ser. Earth Environ. Sci..

[10] Orso C., Moraes M.L., Aristimunha P.C., Della M.P., Butzen F., Krás R.V., Ledur V.S., Gava D., McMaus C.C., Ribeiro A.M.L. (2019). Effect of early feed restriction programs and genetic strain on humoral immune response production in broiler chickens. Poult. Sci..

[11] Carneiro P.R.O., Lunedo R., Fernandez-Alarcon M.F., Baldissera G., Freitas G.G., Macari M. (2019). Effect of different feed restriction programs on the performance and reproductive traits of broiler breeders. Poult. Sci..

[12] Somaia M.A. (2019). The effect of physical feed restriction during the starter period on broilers performance. Int. J. Livest. Prod..

[13] Jahanpour H., Seidavi A., Qotbi A.A.A., Delgado F., Gamboa S. (2014). Effect of intensity and duration of quantitative feed restrictionon broiler caecum microbiota. Indian. J. Anim. Sci..

[14] Zhang T., Ding H., Chen L., Lin Y.Y., Gong Y.S., Pan Z.M., Zhang G.X., Xie K.Z., Dai G.J., Wang J.Y. (2021). Antibiotic-induced dysbiosis of microbiota promotes chicken lipogenesis by altering metabolomics in the cecum. Metabolites.

[15] Xiang H., Gan J.K., Zeng D.S., Li J., Yu H., Zhao H.Q., Yang Y., Tan S.W., Li G., Luo C.W. (2021). Specific microbial taxa and functional capacity contribute to chicken abdominal fat deposition. Front. Microbiol..

[16] Wen C.L., Yan W., Sun C.J., Ji C.L., Zhou Q.Q., Zhang D.X., Zheng J.X., Yang N. (2019). The gut microbiota is largely independent of host genetics in regulating fat deposition in chickens. ISME J..

[17] Cui X.Y., Liu R., Cui H., Zhao G., Zheng M., Li Q., Liu J., Liu Z., Wen J. (2017). Effects of caponization and ovariectomy on objective indices related to meat quality in chickens. Poult. Sci..

[18] Cui X.Y., Gou Z.Y., Fan Q.L., Lin X.J., Wang Y.B., Jiang S.Q., Jiang Z.Y. (2019). Effects of dietary perilla seed oil supplementation on lipid metabolism, meat quality, and fatty acid profiles in Yellow-feathered chickens. Poult. Sci..

[19] Zhang S., Zhong G., Shao D., Wang Q., Hu Y., Wu T.X., Ji C.J., Shi S.R. (2021). Dietary supplementation with *Bacillus subtilis* promotes growth performance of broilers by altering the dominant microbial community. Poult. Sci..

[20] Edgar R.C. (2013). UPARSE: Highly accurate OTU sequences from microbial amplicon reads. Nat. Methods.

[21] Gou Z.Y., Jiang S.Q., Jiang Z.Y., Zheng C.T., Li L., Ruan D., Chen F., Lin X.J. (2016). Effects of high peanut meal with different crude protein level supplemented with amino acids on performance, carcass traits and nitrogen retention of Chinese Yellow broilers. J. Anim. Physiol. Anim. Nutr..

[22] Massuquetto A., Panisson J.C., Marx F.O., Surek D., Maiorka A. (2019). Effect of pelleting and different feeding programs on growth performance, carcass yield, and nutrient digestibility in broiler chickens. Poult. Sci..

[23] Englmaierová M., Skřivan M., Taubner T., Skřivanová V., Čermák L. (2021). Effect of housing system and feed restriction on meat quality of medium-growing chickens. Poult. Sci..

[24] Jahanpour H., Seidavi A., Qotbi A.A.A., Hoven R.V.D., Silva S., Laudadio V., Tufarelli V. (2015). Effects of the level and duration of feeding restriction on carcass components of broilers. Arch. Anim. Breed..

[25] Novel D.J., Ng`ambi J.W., Norris D., Mbajiorgu C.A. (2009). Effect of different feed restriction regimes during the starter stage on productivity and carcass characteristics of male and female Ross 308 broiler chickens. Int. J. Poult. Sci..

[26] Salih R., Tesfaye E., Tamir B., Singh H. (2016). Effects of feed restriction on production performance and carcass characteristics of Koekoek chickens in Ethiopia. Poult. Sci. J..

[27] Li D.Y., Chen H.Q., Mao B.Y., Yang Q., Zhao J.X., Gu Z.N., Zhang H., Chen Y.Q., Chen W. (2017). Microbial biogeography and core microbiota of the rat digestive tract. Sci. Rep..

[28] Sánchez E., Palma G.D., Capilla A., Nova E., Pozo T., Castillejo G., Varea V., Marcos A., Garrote J.A., Polanco I. (2011). Influence of environmental and genetic factors linked to celiac disease risk on infant gut colonization by Bacteroides species. Appl. Environ. Microbiol..

[29] Yang X.J., Zhuang J.Y., Rao K.Q., Li X., Zhao R.Q. (2010). Effect of early feed restriction on hepatic lipid metabolism and expression of lipogenic genes in broiler chickens. Res. Vet. Sci..

[30] Jahanpour H., Seidavi A., Qotbi A.A.A., Payan-Carreira R. (2013). Effects of two levels of quantitative feed restriction for a 7- or 14- days period on broilers blood parameters. Acta Sci. Vet..

[31] Quentin M., Bouvarel I., Berri C., Bihan-Duval E.L., Baéza E., Jégo Y., Picard M. (2003). Growth, carcass composition and meat quality response to dietary concentrations in fast-, medium- and slow-growing commercial broilers. Anim. Res..

[32] Kawasaki T., Iwasaki T., Yamada M., Yoshida T., Watanabe T. (2018). Rapid growth rate results in remarkably hardened breast in broilers during the middle stage of rearing: A biochemical and histopathological study. PLoS ONE.

[33] Jin C.L., Zeng H.R., Gao C.Q., Yan H.C., Tan H.Z., Wang X.Q. (2020). Dietary supplementation with pioglitazone hydrochloride and chromium methionine manipulates lipid metabolism with related genes to improve the intramuscular fat and fatty acid profile of yellow-feathered chickens. J. Sci. Food Agric..

[34] Chodová D., Tůmová E. (2017). Feed restriction and muscle fibre characteristics of pectoralis major in broiler chickens. Sci. Agric. Bohem..

[35] Zhang Z.R., Jiang X.S., Du H.R., Zhu Q., Li X.C., Yang C.W., Liu Y.P. (2012). Characterization of the expression profiles of calpastatin (CAST) gene in chicken. Mol. Biol. Rep..

[36] Piórkowska K., Nowak J., Poltowicz K. (2015). The normalisation of CAPN gene expression in *M. pectoralis* superficialis in broiler lines differing in growth rate and their relationship to breast muscle tenderness. Br. Poult. Sci..

[37] Tong G.Z., Liu X.F., Zhu G., Li X.T., Hai L., Wang W.X. (2020). Study on the relationship between CAPN gene peptide and fat content in sheep muscle. Agric. Biotechnol..

[38] Antonius C., Ginting S.P., Elieser S., Tarigan A., Solehudin S., Budisatria I.G.S., Sari A.P.Z.N.L., Hariyono D.N.H., Maharani D. (2020). The Association of single nucleotide polymorphism (SNP) g.281G > A of CAST gene with meat quality of boerka Goat. Iran. J. Appl. Anim. Sci..

[39] Lippens M., Room G., Groote G.D., Decuypere E. (2000). Early and temporary quantitative food restriction of broiler chickens. 1. Effects on performance characteristics, mortality and meat quality. Br. Poult. Sci..

