# Supplementary material for: Feed Restriction Improves Lipid Metabolism by Changing the Structure of the Cecal Microbial Community and Enhances the Meat Quality and Flavor of Bearded Chickens
Source: Animals (Basel). 2022 Apr 8;12(8):970. doi: 10.3390/ani12080970 (PMC9029254; doi:10.3390/ani12080970)
Supplement: Supplementary file 1 [file animals-12-00970-s001.zip › animals-1644832-supplementary.pdf]

Table S1. Primers for real-time quantitative PCR

Genes	Primers (5′-3′)	Accession Number
FAS	F: AAGCTGAAGGCTGCTGACAA<br>R: CCCTCATCAGTCACCAACAGA	NM_205155.3
ACC	F: TGTGGCACAGAAGAGGGAAT<br>R: CTCTCCCTCGTTTTGCAGGT	NM_205505.1
PPAR-α	F: GCTATCAGCTGTTCACTCGGT<br>R: ACTCATTCAAAAGGAGACAGAGAAC	NM_001001464.1
SREBP-1c	F: GCCCTCGGATGTAGGCTCC<br>R: GGAGGCCAGGGGGTTAAAAG	NM_204126.2
β-actin	F: GAGAAATTGTGCGTGACATCA<br>R: CCTGAACCTCTCATTGCCA	NM_205518

FAS = fatty acid synthase; *ACC* = synthaseacetyl-CoA carboxylase; *PPAR-α* = peroxisome proliferators-activated receptors; *SREBP-1c* = sterol regulatory element binding protein-1c.
